# Spatiotemporal Segregation of Neural Response to Auditory Stimulation: An fMRI Study Using Independent Component Analysis and Frequency-Domain Analysis

**DOI:** 10.1371/journal.pone.0066424

**Published:** 2013-06-18

**Authors:** Natalia Yakunina, Woo Suk Tae, Kang Uk Lee, Sam Soo Kim, Eui-Cheol Nam

**Affiliations:** 1 Department of Otolaryngology, Kangwon National University, School of Medicine, Chuncheon, Republic of Korea; 2 Neuroscience Research Institute, Kangwon National University, School of Medicine, Chuncheon, Republic of Korea; 3 Department of Psychiatry, Kangwon National University, School of Medicine, Chuncheon, Republic of Korea; 4 Department of Radiology, Kangwon National University, School of Medicine, Chuncheon, Republic of Korea; University of Minnesota, United States of America

## Abstract

Although auditory processing has been widely studied with conventional parametric methods, there have been a limited number of independent component analysis (ICA) applications in this area. The purpose of this study was to examine spatiotemporal behavior of brain networks in response to passive auditory stimulation using ICA. Continuous broadband noise was presented binaurally to 19 subjects with normal hearing. ICA was performed to segregate spatial networks, which were subsequently classified according to their temporal relation to the stimulus using power spectrum analysis. Classification of separated networks resulted in 3 stimulus-activated, 9 stimulus-deactivated, 2 stimulus-neutral (stimulus-dependent but not correlated with the stimulation timing), and 2 stimulus-unrelated (fluctuations that did not follow the stimulus cycles) components. As a result of such classification, spatiotemporal subdivisions were observed in a number of cortical structures, namely auditory, cingulate, and sensorimotor cortices, where parts of the same cortical network responded to the stimulus with different temporal patterns. The majority of the classified networks seemed to comprise subparts of the known resting-state networks (RSNs); however, they displayed different temporal behavior in response to the auditory stimulus, indicating stimulus-dependent temporal segregation of RSNs. Only one of nine deactivated networks coincided with the “classic” default-mode network, suggesting the existence of a stimulus-dependent default-mode network, different from that commonly accepted.

## Introduction

Auditory processing has been extensively studied using conventional regression methods such as the general linear model (GLM). Less work has been reported on the application of independent components analysis (ICA) to auditory studies. ICA is a blind-source-separation method that is widely used in fMRI data analysis to identify functionally connected brain networks. It extracts unknown-source blood oxygen level-dependent (BOLD) signals from the known mix of signals. The commonly used spatial ICA decomposes imaging time series into a set of linearly separable, spatially independent components (ICs) and their associated time courses [1]. One of the major problems with hypothesis-based methods is the difficulty to model artifactual signal, which can still be present in the data even after modeling, thus rendering the analysis less than optimal. ICA, on the contrary, is able to isolate networks representing various noise sources, which could facilitate identification of auditory stimulus-induced or unrelated processes [1], [2]. Another distinct feature of ICA, and its advantage as a data-driven approach, is that it does not require an a priori response model of brain activity. This allows avoiding bias towards predicted brain behavior, unlike traditional model-driven analyses. However, the absence of the prior hypothesis makes it difficult to interpret the resulting ICs, separating “meaningful” components reflecting neurobiological and biophysical processes from those reflecting signal artifacts or noise. This issue has been addressed using various approaches in previous studies [1], [3]–[9]. No commonly accepted methodology, however, has been established.

ICA has been increasingly used for investigation of the so-called default-mode network (DMN) [10]. The DMN is commonly defined as a particular set of functionally connected brain regions, including posterior cingulate cortex, parietal and medial prefrontal cortices, that are active at rest but demonstrate consistent deactivation during more demanding cognitive tasks [11], [12]. Although with a certain degree of variability depending on the cognitive load and specifics of the task, previous studies have shown that the overall “resting-state” spatial pattern of the DMN persists across tasks and is thus generally task-independent [11]–[14]. However, a number of studies have demonstrated additional task-induced deactivations that appear outside of the DMN and are different from task to task [15]–[18], suggesting subdivision of the DMN into a task-independent “core” that comprises a common set of regions that consistently show deactivation to various tasks, and a task-specific DMN whose spatial deactivation pattern may vary depending on a given task.

Considering the fact that deactivated areas vary through different tasks, task-induced deactivations may contain significant information and probably should be studied along with task-specific activations to supplement each other’s inferences and provide a more thorough interpretation of results. The same applies for activations following the task or the stimulation with a temporal delay, mostly overlooked by conventional analyses. The goal of the present fMRI study was to investigate spatial characteristics and temporal behavior of neural networks in relation to an auditory stimulus. Along with areas directly activated by the stimulus, we aimed to detect deactivated regions, regions activated by the stimulus with a time lag (following the stimulus waveform with a phase shift), as well as regions unaffected by the stimulus. Spatial ICA was applied to extract multiple brain networks. For the interpretation of the resulting components, we employed the idea of using the stimulus timing frequency as a tool to determine the relation of each network to the stimulus [9]. However, rather than use it to merely determine the degree of task-relatedness of a component, we assumed we could extend it further into classification of the resulting components into four groups using correlation analysis: stimulus-activated (stimulus-dependent fluctuations positively correlated with the stimulus cycles; areas of direct response to the stimulus), stimulus-deactivated (stimulus-dependent fluctuations negatively correlated with the stimulus cycles; areas of DMN evoked by the stimulus), stimulus-neutral (fluctuations induced by, but not correlated with the stimulation, i.e. time-lagged activations), and stimulus-unrelated (fluctuations not following the stimulation). We presumed that such explicit subdivision of the independent networks would possibly reveal temporal segregations in various brain structures in response to the stimulus. We hypothesized that the stimulus-dependent DMN would differ from the classic task-independent DMN. Classical GLM analysis was additionally performed to compare the ICA-based technique employed in this study with conventional methodology.

## Methods

### Subjects

Nineteen healthy subjects (age 24.1±2.22 (SD) years, all right-handed, 15 male) with normal hearing (<20 dB hearing level in a standard audiological frequency range of 250–8000 Hz) participated in this study. Before the fMRI session, all subjects underwent pure-tone audiometry and were examined for loudness discomfort level and dynamic range; subjects who met the criteria of hyperacusis were excluded [19]. Subjects had no known neurological or neuropsychological disorders.

This study was approved by the Institutional Review Board of Kangwon National University Hospital. All subjects gave written informed consent before participation in this study.

### Acoustic Stimulation

Before the experiment, the hearing level and intensity of the sound stimulus for every subject was determined individually using an in-house sound measurement system in the scanner room with the coolant pump turned off. The sound measurement system was a nonmetallic optical microphone integrated in a headphone, which detected the sound intensity in the ear during imaging on a real-time basis and simultaneously provided passive protection against the scanner noise [20]. The lowest intensity sound a subject could hear was defined as the hearing threshold for each ear. Auditory stimuli (continuous broadband white noise with frequency range 0.032–16 kHz) were delivered binaurally at a sensation level of 70 dB above the measured hearing threshold. Sound stimuli were alternately turned on for 32 s and off for 30–36 s (mean 34.4± SD 2.47). Stimulus presentation onset was delayed for 0, 4, or 6 s relative to the first acquired functional image of each run to sample different time points on the hemodynamic response. Each scanning run consisted of four sound-on/−off alternations. Every subject went through one functional session consisting of nine runs. For communication with the operator and emergency situations, a hand-held nurse call button was given to all subjects. Subjects were instructed to stay alert and pay attention to the stimuli.

### Data Acquisition

Subjects were placed in a supine position in the bore of a 1.5-T MRI scanner (Philips Intera; Philips Medical Systems, Best, The Netherlands) equipped with a six-channel Philips SENSE head coil. Sagittal 3D T1-weighted high-resolution structural images of the whole brain were acquired for anatomical orientation (TR 10.20 ms; TE 3.9 ms; FA 8°; matrix 256×256×170; slice thickness 1.0 mm, no gap; FOV 240 mm; voxel size 0.938×0.938×1.0 mm). The brain slices for functional imaging were selected based on the anatomical images. To shorten the acquisition time to reduce the scanner noise duration, ten parallel near-coronal slices were selected covering the areas of interest (brainstem, thalamus, and temporal lobes). The third-posterior-most slice was positioned to intersect the inferior colliculi and the cochlear nuclei. T2*-weighted functional images were acquired using a gradient echo-planar imaging sequence (TE 35 ms; FA 90°; slice thickness 5 mm, no gap; matrix 128×128×10; SENSE acceleration factor R = 2.0; FOV 200 mm; voxel size 1.56×1.56×5.0 mm). Ten selected slices were acquired in the anterior-to-posterior direction (TA = 1.1 s) every 8 s in a sparse acquisition paradigm. Each subject underwent one imaging session consisting of nine runs of 34 acquisitions each; the total number of EPI volumes equaled 306 per subject. The scanner coolant pump was turned off during imaging to reduce the ambient noise level.

### Data Preprocessing

Preprocessing and general linear model analysis were performed using SPM2 (Wellcome Department of Cognitive Neurology, Institute of Neurology, University College London, UK) in the MATLAB 7.0 programming environment (MathWorks, Inc., Natick MA). In each run, the functional images were corrected for head motion, co-registered, normalized to the standard Montreal Neurological Institute (MNI) T1 template, and spatially smoothed with an 8-mm isotropic Gaussian kernel.

### Independent Component Analysis

Spatial ICA was performed using Group ICA in the fMRI toolbox (GIFT v1.3f) [21] for each of the 19 subjects to decompose individual data into 40 components using the Infomax algorithm [2]. For further noise removal, individual ICA results were temporally correlated with each subject’s six head-motion parameters (three translations and three rotations), obtained from the realignment process, and spatially correlated with the cerebrospinal fluid (CSF) template created from each subject’s T1 image. The four most correlated components were selected for each artifact parameter; after elimination of repeatedly found components, a total of 20 “noisy” components for each subject were selected. They were subsequently removed from the original functional EPI data of every individual.

It has been shown that an ICA order of 70±10 components provides the most detailed evaluation of brain networks as compared to low model orders (≤20) that only allow for a general picture of large-scale networks [22]. Considering the partial brain coverage in this study, the order of 40 components was chosen as the most optimal for the intended spatiotemporal segregation. Decompositions with a larger order of separated components (50 and 60) and removal of different numbers of “noisy” components were tried as well, but they were rejected because they did not affect significant components and only resulted in additional noisy components by splitting CSF or motion artifacts into smaller networks. Such components were judged as artifactual because of their tendency to group with CSF spaces, border with brain edges or form a spatially scattered pattern.

The new sets of “denoised” function data of 19 subjects were entered into group ICA. All data were first reduced to 40 principal components; after that, the reduced data were concatenated in time to form a single group-level dataset, and again reduced to 40 components [23]. Independent components (ICs) were extracted using the InfoMax algorithm. After ICA estimation, the components’ time courses and maps were back-reconstructed, resulting in ordered matched sets of individual subjects’ components with corresponding time courses. Group statistical maps of each component were obtained by performing a voxel-wise one-sample *t*–test on these individual independent component maps and then thresholded at false discovery rate (FDR) corrected *q* <0.01. 40 resulting group ICs were subsequently examined visually to determine their neural or artifactual nature. Two independent experts performed visual inspection based upon a procedure described by Kelly et al. [24].

### Frequency Domain Analysis

Fast Fourier transform (FFT) was performed on each time course, averaged across subjects, to obtain its frequency power spectrum. Before performing FFT, a Butterworth high-pass filter was applied to all time courses, with the lower cutoff frequency set to 0.0074 Hz (136 s). The significance of the spectral peaks at the frequencies of interest was tested using a procedure for finding periodic sequences in time series described by Ahdesmäki et al. [25] at *p*<0.05 after Bonferroni correction for the number of tested components and the number of frequency bins. Bonferroni correction was performed by multiplying *p*-values by the number of tested components ( = 16) and the number of frequency bins ( = 135, calculated as the frequency range (0.0625–0.0074 = 0.0551 Hz) multiplied by the number of FFT points (306) divided by the sampling rate (0.125 Hz)). For correlation analysis, time courses were averaged over a single run. The stimulus presentation timing (SPT) waveform was obtained by convolving the stimulus boxcar function with the hemodynamic response function, and then subsampling it at a 8 s interval. Spearman correlation between component time courses and SPT was performed to determine the degree of correlation of corresponding components with the stimulus. Correlations were considered significant at FDR corrected *q* <0.05.

### Classification of the Components

A three-step methodology was used to determine the degree of relation to the stimulus for each component: (1) classification as grey matter (GM) activation based upon visual inspection; (2) power spectrum peak that matched the SPT fundamental frequency or its higher harmonics; and (3) time course significantly correlated with SPT. GM components that had a SPT-matching fundamental frequency were broadly defined as stimulus-related. Among them, components significantly correlated with the stimulus waveform were defined as stimulus-activated if the correlation was positive and stimulus-deactivated if it was negative. Stimulus-related components not significantly correlated with SPT were defined as stimulus-neutral. Components that satisfied only the first condition (i.e., activations in grey matter not following the stimulus waveform) were considered stimulus-unrelated. Components that were classified as artifactual but had a SPT-matching fundamental frequency were defined as stimulus-related non-GM components. The rest of the components were not included in the further analysis.

### General Linear Model Analysis

Multiple regression analysis was performed for each subject. A waveform representing stimulus periods was added as a covariate for the BOLD signal activation. To filter the low-frequency signal drifts of functional data, baseline, linear, and parabolic regressors were included as nuisance variables in the model. Subsequently, a secondary “random effects” analysis was performed by running a one-sample t–test on contrast images calculated from the individual analyses of all subjects. The resulting t–maps were thresholded at FDR corrected q <0.05.

## Results

### Classification of the Components

Removal of “noisy” ICs resulted in more clear separation of subcortical nuclei in the auditory component, reduction of noisy clusters and overall increased signal. By visual inspection, sixteen components were considered grey matter components, because they were found in clusters that matched well with particular gray matter structures rather than being diffusely scattered across large regions or found in the periphery, or matched with previously described networks [26]–[28]. Twenty-four components were classified as artifactual based upon their spotty scattered spatial pattern, tendency to border with brain edges or cluster with CSF compartments, or strong activation corresponding to locations of large vessels.

**Figure 1:**
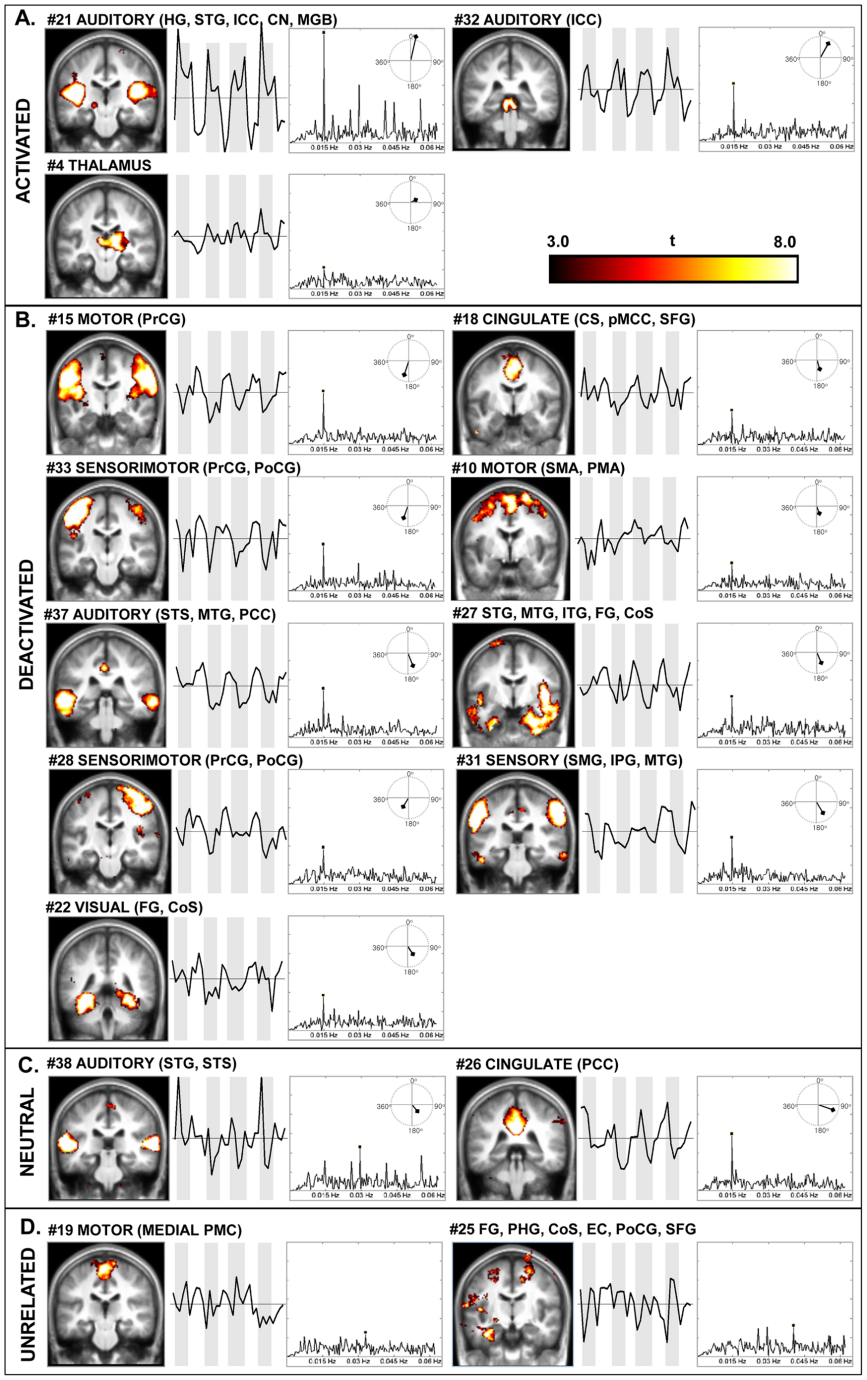
Spatial maps, time courses, and FFT plots of the classified grey matter components. (**A**) Stimulus-activated, (**B**) stimulus-deactivated, (**C**) stimulus-neutral, and (**D**) stimulus-unrelated components. Spatial maps are thresholded at *q* <0.01 FDR corrected and displayed at the most informative coronal slice in neurological convention (the left side of the image corresponds to the left side of the brain). Time courses are averaged across subjects and runs. Grey bars represent stimulus-on periods; the length of bars is fixed for all plots (−0.5 to 0.5 in z-scores). FFT plots are presented on a linear scale; the vertical axis represents the unit amplitude of the spectrum (the axis length is fixed at 0 to 0.15 for all plots). The spectral peak for each component is marked with a black dot. The polar plots depict the difference of the phase at the stimulus presentation frequency from the phase of the stimulus cycles in degrees; the length of the arrow represents the magnitude of the spectrum at the given frequency. CN: cochlear nucleus; CS: cingulate sulcus; CoS: collateral sulcus; EC: entorhinal cortex; FG: fusiform gyrus; HG: Heschl’s gyrus; ICC: inferior colliculus; IPG: inferior parietal gyrus; ITG: inferior temporal gyrus; MGB: medial geniculate body; MTG: middle temporal gyrus; PHG: parahippocampal gyrus; PCC: posterior cingulate cortex; PMA: premotor area; PMC: primary motor cortex; pMCC: posterior midcingulate cortex; PoCG: postcentral gyrus; PrCG: precentral gyrus; SFG: superior frontal gyrus; SMA: supplementary motor area; SMG: supramarginal gyrus; STG: superior temporal gyrus; STS: superior temporal sulcus.

FFT and correlation with SPT were conducted for the time courses of all the components. Stimulus timing frequency was used as a tool to show the relation of each component to the stimulus [9]. Fourteen of 16 grey matter components showed a spectral peak at the fundamental frequency of the stimulus cycles (0.01471 Hz), or its second harmonic (0.02942 Hz) (Table 1). Of these 14 ICs, three were positively correlated with SPT and were defined as stimulus-activated, nine were negatively correlated and defined as stimulus-deactivated, and two did not show significant correlation with stimulus timing and were defined as stimulus-neutral components. The remaining two ICs had a spectral peak frequency that did not match SPT and were hence classified as stimulus-unrelated (Figure 1).

**Table 1:** Temporal and spatial characteristics of the grey matter components.

IC#	r_SPT_	q_SPT_	f (Hz)	Ampl.	Phase (^o^)	Phase diff.	Maxima	Location
**Stimulus-activated**								
21	0.79	0	0.01471	0.145	−56.5	12.8	−42, −24, 10	HG, STS, ICC, CN, MGB
32	0.82	0	0.01471	0.076	−40.2	29.1	−6, −32, −8	ICC
4	0.42	0.01	0.01471	0.028	−6.5	62.9	22, −6, −8	thalamus (mediodorsal)
**Stimulus-deactivated**								
15	−0.87	0	0.01471	0.070	127.8	197.1	64, −12, 32	PrCG
33	−0.78	0	0.01471	0.062	129.3	198.7	−34, −26, 70	PrG, PoCG (left)
37	−0.70	0	0.01471	0.064	88.8	158.1	−60, −34, −12	MTG, STS, PCC
28	−0.58	0	0.01471	0.048	140.8	210.2	44, −26, 48	PrG, PoCG (right)
22	−0.54	0	0.01471	0.046	76.4	145.7	−36, −42, −24	FG, CoS
18	−0.49	0.01	0.01471	0.045	92.7	162.1	4, −18, 52	pMCC, CiS, medial SFG, SMG
10	−0.47	0.01	0.01471	0.037	89.3	158.7	24, −4, 74	SMA, PMA
27	−0.45	0.01	0.01471	0.054	86.5	155.8	30, −8, −38	STG, MTG, ITG, FG, EC, PHG, CoS, hi (right)
31	−0.45	0.01	0.01471	0.061	79.8	149.1	60, −28, 42	SMG, IPG, ITG
**Stimulus-neutral**								
38	−0.07	0.71	0.02941	0.040	70.4	139.7	−54, −20, 4	HG, STS, STG
26	−0.08	0.71	0.01471	0.075	40.1	109.5	4, −32, 30	PCC
**Stimulus-unrelated**								
19	–	–	0.03309	–	–	–	−4, −22, 66	medial PMC
25	–	–	0.04044	–	–	–	−36, −14, −28	FG, PHG, CoS, EC, PoCG, PHG

SPT*:* stimulus presentation timing waveform; r_SPT_: correlation coefficient with SPT; q_SPT_: FDR corrected *q*-value for correlation with SPT; *f*: spectral peak frequency; Ampl.: amplitude of the spectrum at the SPT frequency; Phase: phase of the spectrum at the SPT frequency in degrees; Phase diff.: difference from the SPT phase in degrees; Maxima: coordinates of maxima voxel in MNI space.

CN: cochlear nucleus; CS: cingulate sulcus; CoS: collateral sulcus; EC: entorhinal cortex; FG: fusiform gyrus; HG: Heschl’s gyrus; hi: hippocampus; ICC: inferior colliculus; IPG: inferior parietal gyrus; ITG: inferior temporal gyrus; MGB: medial geniculate body; MTG: middle temporal gyrus; PCC: posterior cingulate cortex; PHG: parahippocampal gyrus; PMA: premotor area; PMC: primary motor cortex; pMCC: posterior midcingulate cortex; PoCG: postcentral gyrus; PrCG: precentral gyrus; SFG: superior frontal gyrus; SMA: supplementary motor area; SMG: supramarginal gyrus; STG: superior temporal gyrus; STS: superior temporal sulcus.

The first of the three stimulus-activated networks, IC 32, showed strong activation in both inferior colliculi. The second highly correlated network, IC 21, comprised the primary and secondary auditory cortices as well as subcortical auditory centers. The last network, IC 4, consisted of the mediodorsal thalamus bilaterally, extending on the right to the lateral dorsal, lateral posterior, ventral lateral, and ventral posterolateral thalamus as well as to the right lateral geniculate body and red nucleus.

Among deactivated networks, IC 15 encompassed the lateral motor cortex. IC 37 embraced the middle temporal gyrus (MTG) and the posterior cingulate cortex (PCC). ICs 33 and 28 both depicted strongly lateralized sensorimotor networks on the left (IC 33) and on the right (IC 28). A somatosensory network was also presented by IC 31, which showed activity in the supramarginal gyrus and inferior parietal gyrus bilaterally. IC 22 showed deactivation in the bilateral fusiform gyrus (FG). IC 10 contained the posterior division of the superior frontal gyrus and extended into the posterior middle frontal gyrus, encompassing the supplementary motor area and the premotor cortex bilaterally. IC 27 comprised a wide bilateral area in the temporal lobes, with much greater spatial extent in the right lobe. IC 18 encompassed a network consisting of the posterior middle cingulate cortex, extending into the cingulate sulcus and the medial superior frontal gyrus and bilaterally in the ventral part of supramarginal gyrus.

The stimulus-neutral group consisted of IC 26, which comprised a single region in the PCC, and IC 38, which embraced both the primary and secondary auditory cortices.

Among stimulus-unrelated components, IC 19 included the medial primary motor cortex. IC 25 was a lateralized network in the left hemisphere, which extended across the parahippocampal gyrus, the collateral sulcus, the entorhinal cortex, and anterior part of the fusiform gyrus, additionally including the ventral postcentral gyrus.

Spectrum magnitudes and phases at the SPT frequency were extracted for the stimulus-related components. The highest magnitude of the spectral peak was expectedly shown by the auditory component (IC 21); it was approximately twice as high as the next highest magnitude (IC 32). The auditory component demonstrated the smallest difference from the SPT phase (12.8° = 2.4 s or 3.6% of a single stimulus cycle). The phases of deactivated ICs were shifted forward by 150–210° relative to that of the stimulus cycles (Table 1). The phases of the components in activated and deactivated subgroups differed from the SPT phase not more than 30° (i.e. 5.7 s or 8.3% of a stimulus cycle) in either direction, counting from 0° for activations and 180° for deactivations. The phases of two stimulus-neutral components were fairly out of that range (ICs 38 and 26). The only exception was IC 4 (thalamus); although it was classified as activated, its phase differed from that of SPT by 62.9°. However, its correlation was the weakest and the peak at the SPT frequency was the lowest among all stimulus-related components.

The standard deviation of ICs’ time courses was calculated to see how much time courses of the stimulus-unrelated components reduced their power after averaging comparing to the stimulus-related components. Mean SD of all averaged time courses was 0.270±0.036, range 0.235–0.381 (the highest SD was that of IC 21 representing the auditory network), with stimulus-unrelated IC 19’s SD = 0.258, and IC 25’s SD = 0.281. All individual time courses had SD = 1 since they were converted to z-scores.

**Figure 2:**
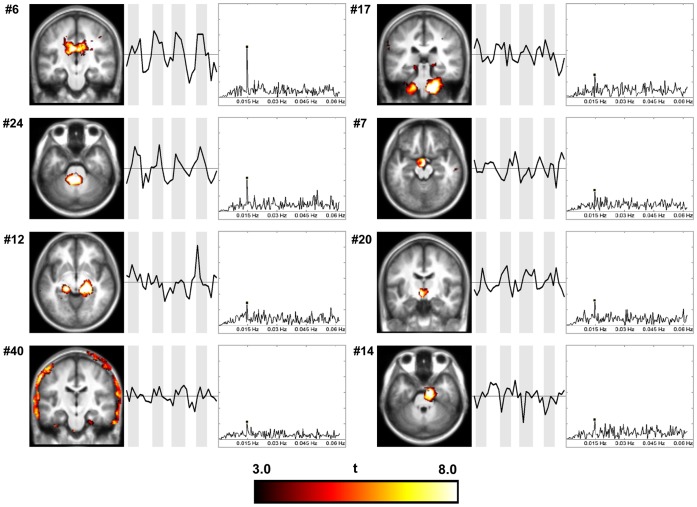
Spatial maps, time courses, and FFT plots of stimulus-related non-GM components. Spatial maps are displayed at the most informative coronal slice in neurological convention (the left side of the image corresponds to the left side of the brain).

Among components classified as artifactual, eight ICs showed a spectral peak at the SPT frequency and were defined as stimulus-related non-GM components (Table 2). All of these components, except one obviously related to the head motion (IC 40), showed activity localized around CSF compartments or large blood vessels, mostly in the brainstem (Figure 2).

**Table 2:** Temporal and spatial characteristics of the non-grey matter components.

IC#	f (Hz)	Maxima	Location
**Stimulus-related non-GM components**			
6	0.01471	−8, −32, 24	CSF (lateral ventricles)
24	0.01471	4, −40, −36	CSF (cerebral aqueduct)
40	0.01471	−64, −14, 46	motion (periphery of the brain)
20	0.01471	−4, −16, −2	CSF (third ventricle)
17	0.01471	16, −24, −44	CSF, BVN (prepontine cistern, basilar artery)
14	0.01471	12, −8, −30	CSF, BVN (prepontine cistern, basilar artery)
7	0.01471	0, −8, −20	CSF (chiasmatic cictern)
12	0.01471	14, −28, −14	CSF (ambient cistern)
**Noise**			
3	0.01225	−16, −20, −32	CSF (prepontine cistern)
2	0.01961	−4, −26, −58	BVN (basilar artery)
8	0.01961	−12, −38, −54	CSF (lateral aperture of fourth ventricle)
23	0.02083	4, −40, −50	CSF (fourth ventricle)
30	0.02083	4, −18, −30	BVN (basilar artery)
16	0.02165	−12, −6, −22	BVN (basal vein)
5	0.02721	8, −38, −24	CSF (ambient cistern)
39	0.02859	4, −32, −58	CSF (premedullary cistern)
35	0.02859	−24, −36, −30	CSF (ambient cistern)
11	0.03554	−2, −22, −2	BVN (internal cerebral vein)
29	0.04330	6, −38, 2	CSF (4th ventricle)
1	0.04804	16, −4, 16	CSF (lateral ventricles)
9	0.05188	−20, −8, 12	BVN (basilar artery)
13	0.05229	6, −38, −66	BVN (vertebral artery)
36	0.05229	6, −24, −44	CSF (prepontine cistern, 3rd ventricle)
34	0.05229	14, −10, −22	BVN (internal carotid artery)

*f*: spectral peak frequency; Maxima: coordinates of maxima voxel in MNI space; BVN: blood vessel network; CSF: cerebrospinal fluid; GM: grey matter.

### ICA vs. GLM Analyses

**Figure 3:**
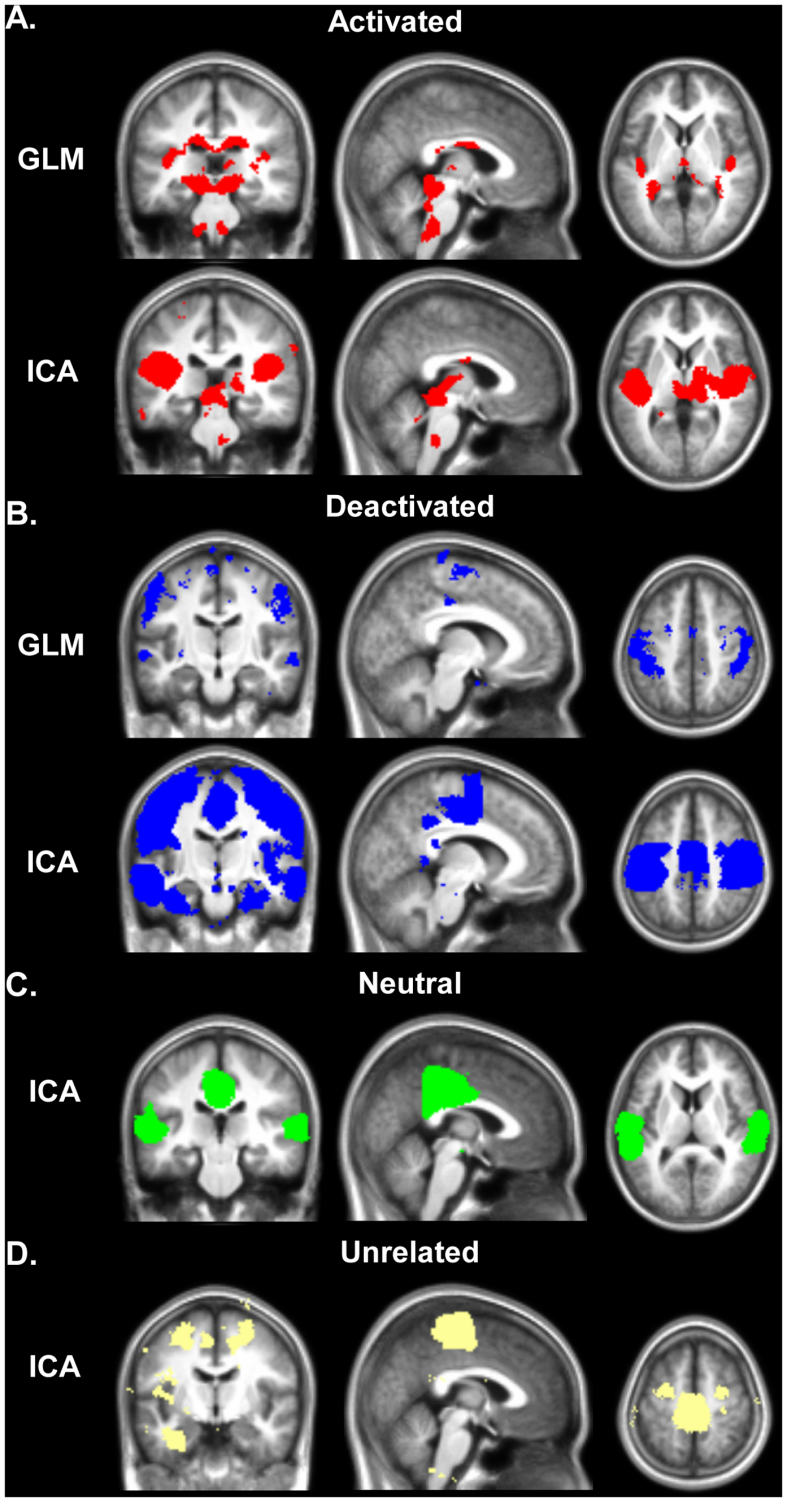
GLM and ICA results. (**A**) Stimulus-activated, (**B**) stimulus-deactivated, (**C**) stimulus-neutral, and (**D**) stimulus-unrelated BOLD signal. GLM spatial maps were obtained using one-sample t-test on contrast images calculated from the individual analyses of all subjects. ICA spatial maps were obtained by overlaying all components in the corresponding classification category. Results are presented on the most representative slices as binary maps thresholded at *p*<0.001 uncorrected for multiple comparisons in neurological convention (the left side of the image corresponds to the left side of the brain).

For consistent comparison, the same threshold was applied to the maps of both ICA and GLM analyses (*p*<0.001 uncorrected for multiple comparisons; Figure 3).

The GLM activation map showed BOLD signals in Heschl’s gyri, the medial geniculate bodies (MGB), inferior colliculi (ICC), and cochlear nuclei (CN) bilaterally, as well as in the corpus callosum and the mediodorsal thalamus. In comparison with ICA, the GLM showed clearer and more obvious activation in the bilateral MGB and CN (Figure 3A).

On the GLM deactivation map, negative activation could be seen bilaterally in the fusiform gyrus, postcentral gyrus, precentral gyrus, medial superior frontal gyrus, anterior superior frontal gyrus, and putamen. A number of regions were detected only by ICA and not by GLM (Figure 3B), such as the posterior cingulate cortex, cingulate sulcus, superior, middle, and inferior temporal gyri, collateral sulcus, parahippocampal gyrus, supramarginal gyrus, and supplementary motor area. All areas of negative BOLD signal changes detected by GLM were detected by ICA. However, the GLM-revealed areas were generally smaller and limited to sub-parts of corresponding ICA-detected areas.

In addition to spatial differences, ICA demonstrated segregation of (de)activation maps into a number of independent networks, each having different time course and phase. ICA also produced stimulus-neutral and unrelated maps that GLM was unable to produce (Figure 3C, D).

## Discussion

This study provides results of independent component analysis applied to auditory stimulation, with subsequent classification of the resulting independent networks according to their relation to the auditory stimulus. 16 of 40 components were identified as non-artifactual grey matter activations and further classified into stimulus-related, that included stimulus-activated, deactivated, and neutral subdivisions, and stimulus-unrelated groups.

### Spatiotemporal Subdivisions in Auditory, Cingulate and Sensorimotor Networks

**Figure 4:**
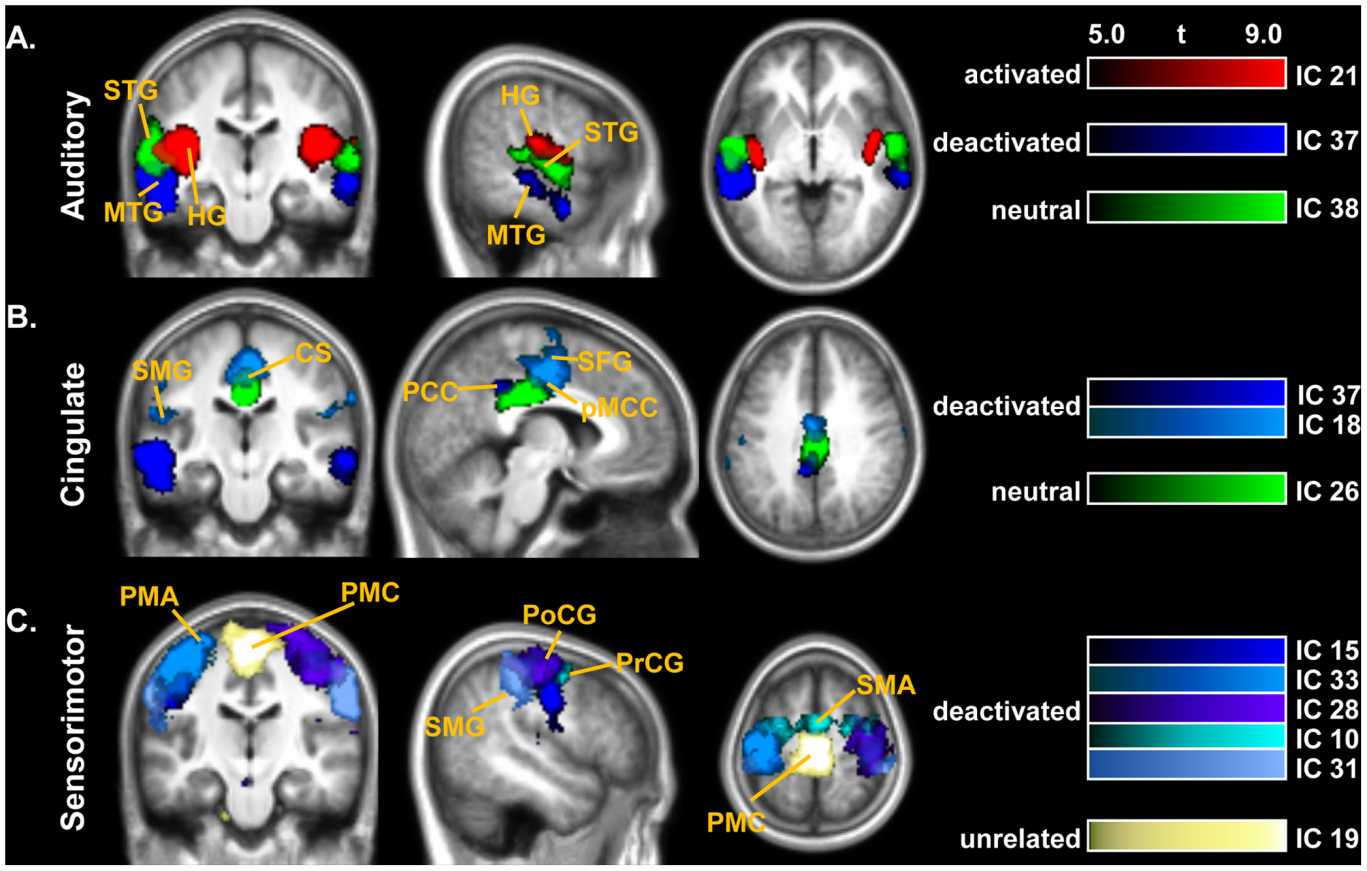
Spatiotemporal segregations in various cortical networks. (**A**) Auditory networks in the temporal lobes, (B) cingulate networks, (C) sensorimotor networks. Spatial maps are displayed in neurological convention (the left side of the image corresponds to the left side of the brain). CS: cingulate sulcus; HG: Heschl’s gyrus; MTG: middle temporal gyrus; PCC: posterior cingulate cortex; PMA: premotor area; PMC: primary motor cortex; pMCC: posterior midcingulate cortex; PoCG: postcentral gyrus; PrCG: precentral gyrus; SFG: superior frontal gyrus; SMA: supplementary motor area; SMG: supramarginal gyrus; STG: superior temporal gyrus.

Spatiotemporal segregations were observed within several cortical networks as a result of classification (Figure 4). Three different stimulus-related networks in the temporal lobes displayed close spatial interconnection but different temporal relation to the stimulus: stimulus-activated Heschl’s gyrus (HG) and posteromedial superior temporal gyrus (STG) (BA 41/42, IC 21), stimulus-neutral lateral STG and superior temporal sulcus (BA 22, IC 38), and stimulus-deactivated MTG (BA 21, IC 37) (Figure 4A). BA 41 and 42 are the locations of the primary and secondary auditory cortex; as expected, these areas were activated in our study, showing the second highest correlation with the stimulus cycles after ICC and the tallest spectral peak at its frequency among all components.

BA 22 is a part of auditory association cortex, which is believed to be involved in word processing, language perception, and integration of auditory and visual information [29], [30]. Additionally, BA 22 has been previously reported to be part of the classic DMN [31]–[33]. This network’s time course showed one full oscillation within every period of either sound-on or sound-off, making it neither activated nor deactivated but neutral. Secondary auditory areas have been reported to have a longer hemodynamic delay comparing to primary regions (∼4–5 s as compared to ∼3 s for the primary auditory cortex) [34], [35], which could be thought to have contributed to the lack of correlation of this component with the stimulus. Such difference, however, would not be reflected in the time courses due to the long TR employed in this study. In addition, long stimulus presentation blocks would render such difference insignificant. Therefore, the neutrality of this network to the stimulus is due to its inherent properties rather than the hemodynamic delay difference**.** The response pattern of a cortical area, auditory in particular, depends on the type of the stimulus (noise/music/speech), duration of the stimulus, its characteristics, repetition time, and other factors. The continuous meaningless white noise was used as a stimulus in this study in order not to evoke emotional or higher cognitive responses associated with the auditory stimulation. Although activations of the secondary auditory cortex to noise have been occasionally reported, this area is mostly activated by meaningful sounds such as music or speech [29], [30], [36]. Cortical activation to the continuous noise is known to be confined to the primary auditory cortex, and even activation of the primary cortex can be partially suppressed by the prolonged stimulation with noise, a phenomenon known as auditory adaptation or fatigue [37], [38]. Therefore, it is not surprising to observe neutral or deactivated areas along with activated regions in the same auditory system in response to the noise stimulus. In addition, it has been shown that the response waveshape of the auditory cortex, especially higher-order regions, tends to shift from sustained to phasic with the increase of sound level and sound repetition rate, showing strong responses to both sound onset and offset [39]–[41]. Such biphasic responses can be obviously seen in the auditory areas in our study (HG, IC 21; ICC, IC 32; and STG, IC 38). For IC 38, it could have been the reason for the spectral peak at the second harmonic of the SPT frequency, and the resulting neutrality of this component.

BA 21 has also been referred to as a part of auditory association cortex; it has been consistently reported to have a role in processing of language, demonstrating activity in response to words, syllables, or, with less intensity, to tones [42]–[44]. In our study it was separated into one network with a cluster in the PCC (BA 23). One possible explanation of deactivation of MTG/PCC network is that it relates to the DMN; both MTG and PCC have been indicated to be a part of the classic DMN in recent meta-analyses [33], [45]. An alternative interpretation of their activation in the absence of the stimulus might be participation in auditory processing. There have been reports of neural inputs from auditory association cortex to the area 23 of caudal cingulate cortex and retrosplenial cortex in primates [46]–[48]. Thus, activity in MTG/PCC might indicate the latter stage of processing of the perceived auditory stimulus after receiving input from primary auditory areas. The subsequent deactivation during stimulus-on periods could possibly be explained by one of the existing theories on the negative BOLD response cause, such as hemodynamic “blood stealing” [49], [50] or “blood sharing” concepts [51], or neuronal activity suppression [52], [53]. Such subdivision in time within the auditory cortex might be evidence of the temporal (time-related) hierarchical organization of the human auditory system.

Another spatial overlap of several independent networks was observed in the cingulate cortex (Figure 4B). The deactivation of the cluster in the PCC along with MTG was described above (IC 37). PCC is a major default mode center; it has been shown to be the only DMN node functionally connected with all other DMN centers [54]. However, in our experiment a bigger area covering the entire PCC did not exhibit any correlation with the stimulus timing, although having the same fluctuation frequency (stimulus-neutral IC 26). Another network showed deactivation in the region overlapping with IC 26 in the posterior midcingulate cortex (pMCC) and extending into the cingulate sulcus and the posteromedial division of the superior frontal gyrus (IC 18). This network also contained the supramarginal gyrus (SMG). Both pMCC and SMG have been shown to deactivate during a modified Stroop task, with the increased deactivation following the increase of task demand [16]. Midcingulate cortex deactivation was also observed during a verbal emotional task [55]. The functional segregation of the cingulate cortex has been discussed in numerous studies. pMCC is believed to be central to skeletomotor regulations and involved in semantic and language tasks, and PCC seems to play important role in relation with visuospatial orientation and self-reflection [56]–[58]. Different parts of the cingulate gyrus have shown activation to various auditory tasks with different task load, with PCC activating even to simple auditory stimulation [59]–[62]. Although the direct activation was not observed in our study, the activity in this cortical area was modulated by passive auditory stimulation, with different cingulate subparts time-locked with the stimulus with different phases, which adds to the notion of multifunctionality of the cingulate cortex subparts [56]–[58].

Six other ICs (15, 33, 28, 31, 10, 19) showed different parts of a sensorimotor area (Figure 4C). Among them, five networks comprising together lateral primary motor and somatosensory areas along with supplementary motor and premotor areas were deactivated by the stimulus. Their phases lied within an approximate range of 80°–140° (Table 1). Although all these networks were classified as deactivated, they displayed different temporal behavior indicating their heterogeneity. Another part of the motor area, medial primary motor cortex was not affected by the stimulus. Together these networks comprise a single sensorimotor resting-state network; its interaction with the stimulus is described in the next section.

### The Identified Components Coincide with the “Resting-state Networks”

All independent brain networks identified in this study comprise parts of previously identified, so-called resting-state networks (RSNs), or brain regions showing coherent low-frequency (0.01–0.08 Hz) fluctuations at rest [31], [63], [64]. Most of the previous resting-state studies were done with continuous sampling (TR = 2–3 s) and thus without removal of scanner background noise effect. Langers and van Dijk explored the effect of scanner noise on intrinsic connectivity networks and concluded that scanner noise from continuous sampling disturbs RSNs more than that of sparse sampling [65]. Gaab et al. demonstrated that scanner noise suppresses the core DMN and suggested a nonlinear influence of the scanner noise on the default mode of brain function [66]. Therefore, the true “resting” nature of previous RSN studies is arguable. We will hence refer to “alleged resting-state networks” in discussing the existing studies, since the effect of scanner background noise was minimized in our study and direct comparison with the results of scanner noise-contaminated studies might not be appropriate.

ICs 21 (posteromedial STG), 4 (thalamus), and 38 (lateral STG) together comprise a single auditory RSN, previously identified in numerous studies [26]–[28]. Six other ICs (15, 33, 28, 31, 10, 19) showed different parts of a sensorimotor area that was previously reported as a single RSN [27], [67]. A recent extensive fMRI study with higher ICA decomposition order identified six different sensorimotor RSNs coinciding with our six networks [26]. IC 18 represents another sensorimotor network consisting of the pMCC and SMG, regions previously reported to be deactivated during task [16]. The pMCC was shown to have strong functional connectivity to the sensorimotor cortex during rest [68]. IC 26 (PCC) and IC 37 (MTG, PCC) represent parts of the classic DMN [33], [45]. The network of IC 22 (FG) is the only network that was not clearly identified among RSNs in previous studies. The right FG was positively correlated with the PCC at rest [69], and the left FG was reported as part of task-induced deactivation network [70]. Another study found consistently increased regional homogeneity in the bilateral FG, suggesting its resting-state activity [71]. Therefore, although not always determinate, this area might be a part of one of the RSNs. ICs 27 and 25 were not considered due to their vague and scattered spatial constitution; IC 32, representing brainstem auditory nuclei (ICC), was not considered due to its subcortical location.

It was clearly observed that the auditory stimulus affected the activity of various RSNs. However, rather than reacting as a unit, different parts of individual RSNs responded to the stimulus with different timings. First of all this leads to a suggestion that even though a certain brain network exhibits coherent spontaneous activity and behaves as one during rest, it actually consists of a number of separate subregions that demonstrate different temporal behavior during external stimulation. Nearly all RSNs were affected by the stimulus: 12 of 13 networks displayed stimulus-dependent fluctuations, with a frequency matching that of the stimulus cycles. Among them, eight subregions were deactivated by the stimulus, comprising a much vaster spatial map than the neutral or unaffected regions. This means that most RSNs activity seems to be suppressed; a massive reallocation of processing resources to the areas of direct response to the stimulus seems to be required. Thus, even passive attending to a simple sound has a considerable impact on inherent brain activity, modulating its connectivity pattern. It gives evidence that the entire brain is continuously working in order to adapt and respond to the changes in the environment, therefore rendering the localized view of brain function by task-related fMRI insufficient. These findings emphasize the importance of combining task-related and resting-state fMRI to complement the interpretations collected from either one.

A recent auditory fMRI study was performed using sound stimulation and methods very similar to those used in this study [72]. However, the subjects performed a simultaneous active visual task. This might be the main reason for the fact that only five independent networks with the frequency matching that of stimulus cycles were discovered, including an auditory network, as opposed to 14 stimulus-related networks in our study. The active visual task might have induced a withdrawal of the resources from a number of RSNs thus influencing their fluctuation pattern.

### Methodological Considerations

Stimulus-unrelated networks deserve particular attention regarding their temporal behavior. Their time courses reduced amplitude very similarly to the stimulus-driven time courses after averaging, which might indicate a behavior systematically repeating itself from run to run. While these networks might have reflected spontaneous fluctuations that repeated every run, another possibility is that their activity might have been affected or evoked by environmental and/or physiological factors related to the fMRI experiment, such as scanner noise, claustrophobic environment, inability to move, keeping eyes closed during a run, or the auditory stimulus producing non-linear hemodynamic change. One or a combination of the above factors could have triggered run-periodic behavior of these networks. Since there is still a chance that these networks were in some way affected by the auditory stimulation, their absolute “unrelatedness” to the stimulus cannot be claimed; rather, “stimulus-unrelated” in this context means “not directly or not linearly related to the stimulus” as compared to the networks that displayed direct dependence on the stimulation (stimulus-related components).

The present paper defines neutral components as “stimulus-related but not correlated with the stimulation timing”; the two stimulus-neutral components showed extremely low correlation coefficients (−0.07 and −0.08). It should be noted, however, that correlation depends on the signal-to-noise ratio; at higher signal strengths otherwise low correlations could be rendered significant due to the low level of noise, while at a lower signal-to-noise ratio stimulus-related components may become stimulus-neutral due to the loss of correlation.

Correlation with the stimulus timing was taken as a criterion for classification into stimulus-related group because it takes into account all frequencies that constitute a time course, thus being a more unbiased measure of stimulus-relatedness than such characteristics as spectral amplitude and phase (shift), which describe the temporal behavior solely at the particular frequency of interest. It means that even with a high spectral peak at the SPT frequency and a relatively small phase difference, if the contributions of other unrelated frequencies are high enough, the correlation of the component may be insignificant (e.g., IC 26), rendering the given component neutral. And vice versa, if the contribution of the SPT frequency is significantly larger than that of other unrelated frequencies, the component could be significantly correlated even if the spectral peak is rather low (IC 4).

The major limitation of our study lies in its incomplete brain coverage. It was originally designed to help eliminate scanner background noise by minimizing acquisition time [73] and explore only areas of interest. As a result, this strategy limited our final conclusions in relationship to the entire brain. A new whole-brain study is being initiated to validate and extend our conclusions.

Another limitation is the inapplicability of the method employed in this study to random aperiodic designs, since the stimulus should be presented at a fixed rate for subsequent power spectra analysis. In this study, however, we did not use strictly periodic stimulus cycles by jittering the onset of the stimulus (sound-off periods mean 34.4±2.47 s, about 30.9% of a TR). It means that for block paradigms, the stimulation does not have to be strictly periodic; the applied technique can still be used even with a certain amount of variability in stimulus cycles.

### DMN Evoked by Auditory Stimulus

The commonly accepted “classic” DMN includes the precuneus/posterior cingulate cortex, medial prefrontal cortex, and medial, lateral, and inferior parietal cortices [10], [12]. There have been a few investigations into DMN behavior evoked by auditory stimulation, mainly focusing on auditory target-detection tasks [12], [65], [74]–[76]. The DMN demonstrated in these studies generally matched the classic “core” DMN pattern, except for the work of Calhoun et al., who discovered additional deactivation in the right lateral frontal area in the auditory oddball task [17]. No results have been reported on DMN behavior during passive listening to a simple sound.

Nine networks showed deactivation during sound-on and activation during sound-off periods with the SPT-matching fundamental frequency (Figure 3B, bottom panel), indicating they are part of a stimulus-dependent DMN. Within the brain volume studied, only one of nine networks (MTG/PCC, IC 37) matched with the classic DMN. Previous studies found that DMN deactivations increased spatially with increased task demand [12] and that the activity in DMN persisted during passive sensory stimulation (i.e., DMN does not deactivate if the task is not sufficiently challenging) [13], [14]. However, we observed extensive deactivation outside the common DMN in response to passive stimulation with a simple noise without any task demand load.

Although this deactivation is obviously evoked by the auditory stimulus considering its SPT-matching frequency, calling it solely auditory-specific DMN is not possible based upon our experiment alone, since no comparison was done with other types of stimulation. It is a possibility that a similar, or partly similar, network could be deactivated by other sensory stimuli. Nevertheless, we demonstrated that deactivations induced by a simple noise stimulus lie outside of the commonly accepted DMN. Our observations contribute to the previous findings that task- or stimuli-induced deactivations may differ through different tasks or stimuli, and accentuate the significance of exploring stimulus- or task-activated and deactivated networks simultaneously, as both seem to carry important information about brain function. Future studies with full brain coverage and comparison with other types of stimulation are warranted to draw conclusions about the precise structure of the auditory DMN and its spatial and functional relationship with the core DMN.

### Stimulus-dependent Activity Outside of Grey Matter

Eight components representing activity outside of grey matter structures were found to have the same frequency as the stimulus cycles (Figure 2). Their relation to the stimulus cannot be explained by stimulus-induced neuronal response. The coherence with the stimulus of the component related to the head motion (IC 40) can be explained by subjects’ tensing at the sound onset and relaxing after sound offset. All other components were concentrated around CSF or large blood vessels locations. It has been shown that exposure to noise, defined as unwanted sound, at high enough intensities evokes neural and hormonal responses, leading to temporary increases in blood pressure, heart rate, and vasoconstriction [77]–[79]. Continuous broadband white noise could have triggered autonomous stress responses including vasoconstriction through the release of adrenergic agonists, and resulted in cyclic vasomotion following the onset and offset of the noise stimulation [80], that would originate periodic blood supply yielding stimulus-synchronized BOLD fluctuations. Taking into account the dependency between blood flow and CSF pulsation [81], such stimulus-coherent activity could extend into general CSF spaces as well.

Another possibility is the so-called physiological noise, namely fluctuations related to respiratory and cardiac processes. These signals were undersampled in our study which means their fundamental frequency was aliased onto the unknown spectrum, leaving a chance of coinciding with one of the frequencies of interest, especially a higher harmonic. Such fluctuations have been shown to be mostly confined to locations within and near large blood vessels and/or CSF, or the outline of the brain [82]–[84]. Therefore, the activity related to physiological fluctuations would most probably be classified into one of non-GM subgroups, either stimulus-related non-GM group if its frequency matched that of SPT, or just noise.

Although ICA could innately separate noise-related activity, the resulting GM components can still contain a certain number of white matter voxels, which contribute to the temporal characteristics of the components and hence affect their classification pattern. Relating activation of white matter to noise, Formisano et al. demonstrated that ICA performed on only cortical GM results in cleaner time courses [8]. However, a growing number of fMRI studies have been demonstrating activations in white matter, providing evidence that they might depend on the task and be functionally manipulated [85]–[89]. Recent works showed that white matter hemodynamic response characteristics are comparable to those of grey matter with decreased peak response amplitudes [90]. Therefore, white matter activations should not be disregarded when investigating task-related brain activity, and the influence of white matter voxels on the overall independent network’s temporal behavior should be considered along with grey matter contribution.

### Comparison with Regression Analysis

It has been reported that for an auditory oddball paradigm regression-based analyses perform better compared to ICA for analysis of task-related activations in cases when the prior information is accurate [91]. Our results also show that GLM appears to be a solid technique for demonstrating areas of direct activation in response to an auditory stimulus. However, in terms of stimulus-related deactivation, ICA detected all areas revealed by GLM plus additional areas that were not on the GLM map even with lowered threshold. It has been shown previously that, comparing with GLM, ICA is able to detect additional task-related decreases associated with DMN [91].

A major limitation of a model-based approach is that the observed activation patterns merely follow the regressors included in the model, and consequently, hemodynamic responses that are not explicitly specified cannot be detected. In this study, linear regression analysis was performed in a classical manner, strictly following the reference waveform. Thus, it was able to reveal only regions of activation and deactivation directly induced by the stimulus, and not activations following the stimulus with a phase shift (“stimulus-neutral” group revealed by ICA, Figure 3C). To account for such response latency, the use of various additional sets of basic functions as a predictor of interest has been demonstrated, such as temporal derivative of the response function [92], [93], Hilbert transform [94], Fourier transform or a set of sinusoidal functions [95]–[97], or a flexible basis set of response functions “OSORU” [41]. Langers and Melcher employed a combination of sinusoids to explore possible responses in non-auditory areas to auditory stimulation with continuous broadband noise in a paradigm similar to the paradigm in this study [72]. In addition to activation in classical auditory pathway, activations were found in the hippocampus, caudate nucleus, insula, and superior thalamus. The same structures were detected by ICA; however, ICA-revealed networks amounted to much more extensive spatial maps that included structures absent in GLM outcomes, similar to our result for deactivation. Another study reported that ICA revealed additional activation in the primary motor cortex in performing a visual task, which could not be detected by GLM alone [98].

Thus, parametric techniques seem to overlook stimulus-related activations, negative or neutral in particular, which are successfully detected by ICA, even when an a priori response model is altered to account for activity different in phase with the task. A further limitation of regression methods is that they are not capable of revealing “background” activity unrelated with a task at hand (“stimulus-unrelated” group by ICA, Figure 3D). In the meantime, the knowledge of how a certain stimulus or task affects inherent brain activity might be crucial for the ultimate understanding of the overall brain functional framework. By tracking background activity, ICA generally overcomes the limitation of false assumption that all brain processes other than the one of interest are constant between the experiment and control conditions.

Another virtuous feature of ICA is that it is able to differentiate between activities induced by different brain functions that have different dynamics, even when all of them respond to the same stimulation pattern. GLM, on the other hand, if provided only with the stimulus waveform, aggregates all responses that follow the stimulation into a single (de)activation map. Yet, as ICA has demonstrated particularly for negative and neutral activity, such maps actually consist of a number of mutually independent networks, each responding to the stimulus with a different time course and a different phase shift. It signifies that these networks have different functions and thus probably different reasons of their responses to the stimulus, which is impossible to discern using GLM without a priory information about each network’s temporal dynamics. In other words, spatiotemporal segregation of brain response in case when only stimulation information is known in advance is not possible through the use of conventional methods.

Ultimately, no matter how delicately the parameters are assigned, hypothesis-driven confirmatory approaches will always remain biased to a predicted model of response, leaving a chance of missing a relevant signal that does not follow the fit design. ICA, on the other hand, is an utterly flexible approach that is capable of modeling a signal of any complexity, allowing for detection of nontrivial, unpredicted, or unanticipated stimulus-related activity that may not be detectable using conventional approaches of completely model-based analyses.

### Conclusions

Temporal segregation in response to the auditory stimulus was observed in a number of cortical structures including the auditory system. Most of the identified networks related to the stimulus lay outside of the common auditory pathway and comprised sub-parts of the known resting-state networks (RSNs). All except one considered RSN subregions were influenced by the stimulus; their temporal behavior, however, was different. This implies that intrinsic brain behavior is modulated even by passive sound stimulation. The resting-state activity is considerably affected in an attempt to adjust to the external environment, which shows the necessity of exploring task-related changes along with the changes in the resting state behavior. This would allow for more detailed characterizations of brain activity, eventually contributing to a more complete interpretation of the human brain functional architecture.
